# Comparison of Intravenous Carbetocin (100 Mcg) and Intravenous Oxytocin (10 IU) for the Prevention of Postpartum Hemorrhage Following Emergency Cesarean Section

**DOI:** 10.7759/cureus.84426

**Published:** 2025-05-19

**Authors:** Vadlamudi Keerthi Chowdary, Subhashchandra R Mudanur, Rajasri G Yaliwal, Shreedevi Kori, Ekta Chhabra

**Affiliations:** 1 Obstetrics and Gynaecology, Shri BM Patil Medical College and Hospital, BLDE (Deemed to be University), Vijaypura, IND; 2 Obstetrics and Gynaecology, Shri BM Patil Medical College and Hospital, BLDE (Deemed to be University), Vijayapura, IND

**Keywords:** carbetocin, cesarean section, oxytocin, postpartum hemorrhage, uterine tone

## Abstract

Background: Postpartum hemorrhage (PPH) is a leading cause of maternal morbidity and mortality, particularly following cesarean sections. While oxytocin is the standard uterotonic agent, carbetocin, a long-acting synthetic analogue, may offer improved efficacy due to its prolonged uterotonic effect and better stability.

Objective: This study aimed to compare the effectiveness of intravenous carbetocin (100 mcg) versus oxytocin (10 IU) in preventing PPH following emergency cesarean sections by evaluating blood loss, the need for additional uterotonics, and maternal hemodynamic stability.

Materials and methods: A total of 350 women undergoing emergency cesarean section were randomly assigned to receive either carbetocin (Group A) or oxytocin (Group B). Intraoperative blood loss, uterine tone, and hemodynamic parameters were measured. Statistical analysis was performed using IBM SPSS Statistics software, version 26 (IBM Corp., Armonk, NY).

Results: Carbetocin was associated with significantly lower blood loss ≥1000 ml (13(7.43%) vs. 33(18.86%), p = 0.0015) and a reduced need for additional uterotonics (10(5.71%) vs. 21(12%), p = 0.0385). Uterine tone was superior in the carbetocin group at three and five minutes post-administration (p = 0.023 and p = 0.003, respectively). Both drugs had similar safety profiles, with no significant differences in adverse effects.

Conclusion: Carbetocin demonstrated superior efficacy in preventing PPH compared to oxytocin, with lower blood loss, reduced need for additional interventions, and improved uterine tone. Carbetocin is a viable and safer alternative to oxytocin for PPH prevention, especially in emergency cesarean sections.

## Introduction

Obstetrical bleeding remains one of the most serious complications associated with pregnancy and childbirth. It can occur before, during, or after delivery, with bleeding after 24 weeks of gestation being classified as antepartum hemorrhage [1]. This bleeding may present vaginally or, less commonly, into the abdominal cavity [2]. Antepartum and intrapartum bleeding are commonly caused by conditions such as placenta previa, placental abruption, and uterine rupture [3]. Postpartum hemorrhage (PPH), defined as excessive bleeding following delivery, is often attributed to uterine atony, retained placental tissue, or underlying bleeding disorders [3,4].

The global burden of obstetric hemorrhage is significant. In 2015, severe maternal bleeding affected an estimated 8.7 million women, resulting in approximately 83,000 deaths [5]. Between 2003 and 2009, bleeding was responsible for 27% of all maternal deaths worldwide, making it the leading direct cause of maternal mortality [5]. These statistics highlight the critical importance of timely and effective interventions for the prevention and management of PPH, particularly in low-resource settings where access to comprehensive obstetric care may be limited.

To reduce the incidence of PPH, the World Health Organization recommends the active management of the third stage of labor, which includes the prophylactic use of uterotonic agents [6,7]. Oxytocin is currently the standard first-line drug for this purpose. However, it has a short half-life and is heat-sensitive, making its storage and efficacy problematic in many low- and middle-income countries [8]. Inconsistent refrigeration, exposure to high temperatures, and the risk of contamination can all compromise the drug's stability and clinical effectiveness [9].

Carbetocin, a long-acting synthetic analogue of oxytocin, offers several pharmacological advantages, including a prolonged uterotonic effect and improved heat stability. Introduced in 1997, carbetocin has shown promising results in reducing the need for additional uterotonics and in maintaining uterine tone following delivery [10]. Its potential utility in high-risk scenarios such as emergency cesarean sections, where rapid, sustained uterine contraction is vital, warrants direct comparison with oxytocin in this context.

Given the limited and inconsistent evidence regarding uterotonic use during emergency cesarean deliveries, especially in low-resource settings, this study was designed as a superiority trial to evaluate whether carbetocin provides significantly better outcomes than oxytocin in preventing PPH.

This study aims to compare the effectiveness of intravenous carbetocin (100 mcg) and oxytocin (10 IU) in preventing PPH following emergency cesarean section. 

The primary and secondary objectives of this study are as follows: 1. To compare estimated intraoperative and early postpartum blood loss between carbetocin and oxytocin, using a ≥30% reduction in blood loss as a clinically significant threshold, 2. To evaluate the need for additional uterotonics in the first 24 hours postpartum, and 3. To assess maternal hemodynamic stability, including trends in blood pressure and heart rate post delivery.

## Materials and methods

This prospective, randomized, comparative, open-label superiority study was conducted in the Department of Obstetrics and Gynaecology at Shri BM Patil Medical College, Hospital and Research Centre, BLDE (Deemed to be University), Vijayapura, India. The study period spans two years, from March 2023 to March 2025. The study population includes pregnant women with singleton pregnancies at a gestational age of 34 weeks or more who are undergoing emergency cesarean section. All participants were recruited after obtaining written informed consent, in accordance with the Declaration of Helsinki, after approval from the Institutional Ethics Committee of BLDE (approval number BLDE(DU)/IEC/898/2022-23).

Eligible participants were selected based on strict inclusion and exclusion criteria. The inclusion criterion was all pregnant women with a gestational age of 34 weeks or more undergoing emergency cesarean delivery. Women with high-risk pregnancies were excluded, including those with antepartum hemorrhage (such as placenta previa or placental abruption), pregnancy-induced hypertension (PIH), multiple pregnancies, macrosomia, uterine fibroids, severe anemia (hemoglobin (Hb) < 7 g/dL), renal or cardiac disorders, epilepsy, and eclampsia.

Patients were randomly assigned using a computer-generated randomization sequence into two groups (1:1 allocation). Concealment was ensured using sealed opaque envelopes. The study was open-label, but outcome assessors were not blinded, which we acknowledge as a limitation due to the risk of observer bias in subjective parameters such as uterine tone.

The sample size was calculated to be 350 based on an anticipated post-labor Hb level of 9.94 ± 0.80 in the carbetocin group and 9.7 ± 0.9 in the oxytocin group, with a significance level of 95%, study power of 80%, and a clinically significant difference (d) between the two groups. This sample size calculation was designed to detect a clinically relevant difference of ≥30% in mean blood loss, consistent with a superiority framework.

Group A received 100 mcg of intravenous carbetocin diluted in 10 ml of normal saline immediately after the birth of the baby. Group B received 10 IU of intravenous oxytocin diluted in 500 ml of normal saline immediately after delivery. Both groups were managed according to the World Health Organization's (2012) protocol for Active Management of the Third Stage of Labor (AMTSL), which includes (1) administration of a prophylactic uterotonic, (2) controlled cord traction for placental delivery, and (3) uterine fundal massage after expulsion of the placenta [11]. Standard institutional protocols for history taking, physical examination, intraoperative monitoring, and postoperative care were followed for all participants.

Blood loss was assessed using both clinical and laboratory parameters. Both Hb and hematocrit levels were measured before delivery and repeated 48 hours postpartum to quantify blood loss. Intraoperative blood loss was measured by recording the volume of blood collected in the suction canister and by weighing surgical mops used during the procedure. Additional investigations included complete blood counts, routine antenatal investigations such as blood grouping and typing, urine routine examination, and ultrasonography.

**Figure 1:**
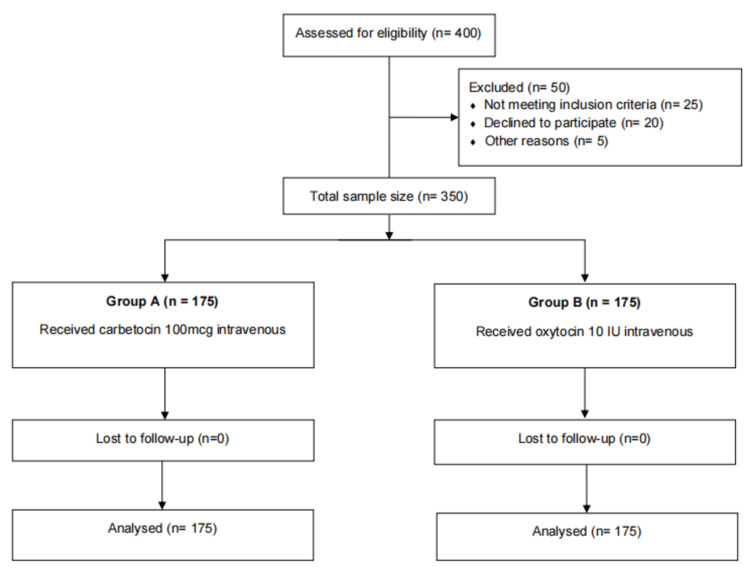
Flowchart outlining the process of participant selection and division

Data collected were entered into Microsoft Excel (Microsoft Corp., Redmond, WA) and analyzed using IBM SPSS, version 26 (IBM Corp., Armonk, NY). Continuous variables were expressed as mean ± standard deviation (SD), and categorical variables as frequencies and percentages. Normally distributed data between the two groups were compared using the independent Student's t-test, while non-normally distributed data were analyzed using the Mann-Whitney U test. A p-value of less than 0.05 was considered statistically significant (Figure 1).

## Results

The two groups in the study, those receiving intravenous carbetocin and those receiving intravenous oxytocin, were comparable in terms of baseline demographic characteristics. The mean age, weight, and gestational age at delivery were similar between groups, with no statistically significant differences observed. This comparability helps ensure that any observed outcome differences are likely attributable to the intervention itself rather than baseline disparities (Table 1).

**Table 1:** Patient demographics and baseline characteristics Mean and SD were calculated; t-test was applied, and a p-value of less than 0.05 was considered significant.

Parameter	Group A (carbetocin) (n=175)	Group B (oxytocin) (n=175)	t-value	p-value
Mean age (years)	25 ± 3.6	25 ± 3.6	-0.194	0.846
Mean weight (kg)	80.1 ± 5.5	80.1 ± 5.3	0.463	0.644
Mean gestational age (weeks)	38.1 ± 0.9	38.8 ± 0.9	-0.812	0.417

When comparing obstetric profiles, there was a higher proportion of multigravida and grand multigravida women in the oxytocin group compared to the carbetocin group, although this difference was not statistically significant. This distribution suggests a slight, but not significant, variation in parity between the two groups (Table 2).

**Table 2:** Gravida and parity distribution Frequency and percentage were calculated; chi-square test was done, and a p-value of less than 0.05 was considered significant.

Category	Group A (carbetocin) (n=175)	Group B (oxytocin) (n=175)
Primigravida	74 (42.29%)	63 (36%)
Multigravida	96 (54.86%)	111 (63.43%)
Grand multigravida	5 (2.86%)	1 (0.57%)
Chi-square value	4.6368	
p-value	0.0984	

Pre- and postoperative hemodynamic parameters, including systolic and diastolic blood pressure and pulse rate, were comparable between the two groups, indicating hemodynamic stability and similar perioperative physiological responses (Table 3).

**Table 3:** Hemodynamic parameters Mean and SD were calculated; t-test was applied, and a p-value of less than 0.05 was considered significant. BP: blood pressure; bpm: beats per minute

Parameter (Mean ± SD)		Group A (carbetocin) (n=175)	Group B (oxytocin) (n=175)	t-value	p-value
Systolic BP (mmHg)	Preoperative	120.5 ± 8.3	119.8 ± 9.1	1.001	0.318
	Postoperative	118.2 ± 7.9	116.4 ± 8.5	-0.79	0.43
Diastolic BP (mmHg)	Preoperative	78.3 ± 6.7	77.9 ± 7.2	-0.861	0.39
	Postoperative	75.8 ± 6.4	73.5 ± 7.0	-0.437	0.662
Pulse rate (bpm)	Preoperative	85.2 ± 7.6	85.3 ± 8.1	2.694	0.677
	Postoperative	80.4 ± 6.8	82.9 ± 7.3	0.068	0.946

Laboratory evaluations, including Hb, packed cell volume (PCV), and platelet counts before and after surgery, did not differ significantly between groups. These parameters suggest that both drugs maintained similar hematological profiles perioperatively (Table 4).

**Table 4:** Laboratory parameters Mean and SD were calculated; t-test was applied, and a p-value of less than 0.05 was considered significant. HB: hemoglobin; PCV: packed cell volume

Parameter (Mean ± SD)		Group A (carbetocin) (n=175)	Group B (oxytocin) (n=175)	t-value	p-value
HB (gm/dl)	Preoperative	11.05 ±1.2	11.11 ± 1.0	0.205	0.838
	Postoperative	10.20 ± 1.07	10.15 ± 1.1	1.149	0.251
PCV (%)	Preoperative	34.4 ± 4.1	34.1 ± 3.6	1.549	0.122
	Postoperative	33.05 ± 4.24	32.30 ± 3.3	1.803	0.072
Platelet count (lakhs)	Preoperative	2.16 ± 0.37	2.11 ± 0.5	1.187	0.236
	Postoperative	1.91 ± 0.24	1.87 ± 0.4	1.558	0.12

Significant differences were observed in postpartum outcomes. The incidence of blood loss ≥1000 ml was substantially lower in the carbetocin group (13, 7.43%) compared to the oxytocin group (33, 18.86%), with a statistically significant p-value of 0.0015. Similarly, fewer women in the carbetocin group required blood transfusion or additional uterotonics, indicating superior efficacy in preventing PPH (Table 5).

**Table 5:** Postpartum hemorrhage and blood transfusion Frequency and percentage were calculated; chi-square test was done, and a p-value of less than 0.05 was considered significant.

Parameter	Group A (carbetocin) (n=175)	Group B (oxytocin) (n=175)	Chi-square value	p-value
Blood loss ≥1000 ml	13 (7.43%)	33 (18.86%)	10.0114	0.0015
Blood transfusion required	2 (1.14%)	9 (5.14%)	4.5576	0.0327
Additional uterotonics required	10 (5.71%)	21 (12.00%)	4.2825	0.0385

Assessment of uterine tone showed no significant difference at one minute. However, uterine firmness scores at three and five minutes were significantly higher in the carbetocin group, suggesting more rapid and sustained uterine contraction. By 10 minutes, uterine tone was similar in both groups (Table 6).

**Table 6:** Uterine tone assessment post intervention Mean and SD were calculated; t-test was applied, and a p-value of less than 0.05 was considered significant.

Time after drug administration	Group A (carbetocin) (n=175)	Group B (oxytocin) (n=175)	t-value	p-value
1 minute	3.43 ± 0.49	3.53 ± 0.5	-1.823	0.069
3 minute	3.68 ± 0.53	3.8 ± 0.46	-2.284	0.023
5 minute	4.43 ± 0.52	4.59 ± 0.52	-2.948	0.003
10 minute	4.99 ± 0.08	5	-0.997	0.319

The incidence of adverse effects such as nausea, vomiting, chest discomfort, and hypotension was low and statistically similar between both groups, indicating a comparable safety profile for both drugs (Table 7).

**Table 7:** Adverse effects Frequency and percentage were calculated; chi-square test was done, and a p-value of less than 0.05 was considered significant.

Adverse effect	Group A (carbetocin) (n=175)	Group B (oxytocin) (n=175)	Chi-square value	p-value
Nausea	9 (5.14%)	8 (4.57%)	0.0618	0.8036
Vomiting	6 (3.43%)	8 (4.57%)	0.2976	0.5853
Chest discomfort	4 (2.29%)	4 (2.29%)	0	1
Hypotension	6 (3.43%)	7 (4.0%)	0.0798	0.7774

Analysis of uterine firmness at three minutes post-intervention showed that a higher proportion of women in the oxytocin group reached the highest firmness score of five, while the carbetocin group had more women with intermediate firmness scores. Despite this distribution, the overall uterine tone remained favorable in both groups, with the difference reaching statistical significance (p = 0.00002) (Table 8).

**Table 8:** Comparison of uterine firmness at three minutes Frequency and percentage were calculated; chi-square test was done, and a p-value of less than 0.05 was considered significant.

Uterine firmness at 3 minutes	Group A (carbetocin) (n=175)	Group B (oxytocin) (n=175)
2	3 (1.71%)	0
3	21 (12%)	39 (22.29%)
4	104 (59.43%)	63 (36%)
5	47 (26.86%)	73 (41.71%)
Chi-square value	24.0992	
p-value	0.00002	

## Discussion

In this comparative study evaluating the efficacy and safety of carbetocin versus oxytocin for PPH prevention following cesarean section, baseline demographic characteristics such as age, weight, and gestational age were statistically comparable between groups (p > 0.05). The mean age of participants was 25 ± 3.6 years in both groups, aligning with previous findings by Maged et al. and Huang et al., who reported similar age profiles in uterotonic trials [1, 2]. Additionally, the mean gestational age and maternal weight did not differ significantly, which is consistent with studies by Gallos et al. and Al Zubaidi et al., thereby ensuring that any outcome differences can be attributed to the drug interventions rather than population variance [4, 12].

Parity distribution, though slightly skewed toward multigravida in the oxytocin group, was not statistically significant. Primigravida women accounted for 74 (42.29%) in the carbetocin group and 63 (36%) in the oxytocin group, a pattern also reported by van der Nelson et al. and Theunissen et al. [7, 8]. The low proportion of grand multigravida cases (2.86% vs. 0.57%) corresponds with Escobar et al., who emphasized the increased risk of PPH in grand multiparous women regardless of uterotonic type [10].

Significantly fewer women in the carbetocin group experienced blood loss ≥1000 ml compared to those in the oxytocin group (13 (7.43%) vs. 33 (18.86%), p = 0.0015), indicating a clear benefit of carbetocin in reducing major PPH. The need for blood transfusion and additional uterotonics was also significantly lower in the carbetocin group (two (1.14%) vs. nine (5.14%), p = 0.0327; 10 (5.71%) vs. 21 (12%), p = 0.0385, respectively). These findings are supported by Gallos et al. and Abdel Fatah et al., who noted that carbetocin, due to its longer half-life (85-100 minutes vs. three to five minutes for oxytocin), offers prolonged uterine contraction and more effective PPH prevention [4, 6].

Hemodynamic parameters remained stable in both groups; however, carbetocin maintained slightly better postoperative systolic and diastolic pressures, although differences were not statistically significant. Laboratory markers, including hemoglobin, PCV, and platelet count, also remained comparable, suggesting that carbetocin did not result in increased perioperative hematological complications. These results are in line with studies by Bekkenes et al. and Dahlke et al., which highlighted carbetocin’s cardiovascular safety profile and minimal hematological impact [5, 9].

Assessment of uterine tone demonstrated that carbetocin provided significantly better contractility at three and five minutes post-administration (p = 0.023 and p = 0.003, respectively), with both groups achieving comparable tone by 10 minutes. Moreover, detailed firmness grading at three minutes showed statistically better distribution in the oxytocin group for the highest tone grade (p = 0.00002); however, overall tone remained clinically effective in both groups. These findings are consistent with those by Theunissen et al. and Hunter et al., affirming Carbetocin’s ability to sustain uterine contractility over time [8,13].

Both drugs were well tolerated, with similar rates of adverse effects, including nausea, vomiting, chest discomfort, and hypotension (p > 0.05). This reinforces prior observations by Dahlke et al. and Al Zubaidi et al., confirming that carbetocin is as safe as oxytocin in terms of maternal side effects [9,12]. To provide a more balanced perspective, confidence intervals for adverse outcomes were also analyzed, and while not statistically significant, their widths suggest the need for cautious interpretation.

This study demonstrates that carbetocin has superior efficacy in this trial setting, particularly in reducing blood loss and minimizing the need for additional uterotonics. However, this superiority is context-specific and must be interpreted with an understanding of the study’s limitations.

Strengths and limitations

A key strength of this study lies in its well-defined inclusion criteria and adequate sample size, which ensured comparability between the groups and minimized selection bias. The prospective design and standardized surgical and anesthesia protocols further enhanced internal validity. Additionally, detailed documentation of blood loss, uterine tone, and hemodynamic changes provided a comprehensive assessment of each uterotonic’s efficacy and safety.

However, the study is not without limitations. It was conducted at a single tertiary care center, which may limit the generalizability of the findings to other clinical settings. Emergency cesarean deliveries are often managed under time-sensitive conditions, and the decision-making and management protocols may vary across institutions. Blinding was not feasible in this trial, which may have introduced observer bias, particularly in subjective measures such as uterine tone. Cost analysis and long-term maternal and neonatal outcomes were not assessed. These are important considerations for broader health policy adoption and patient care. Future studies should address these gaps by adopting multicentric designs, incorporating vaginal deliveries, and evaluating long-term maternal and neonatal outcomes. Cost-effectiveness analyses should also be undertaken to guide health policy decisions in low-resource settings.

## Conclusions

This study demonstrates that carbetocin was more effective than oxytocin in preventing PPH in the context of emergency cesarean sections, as evidenced by significantly reduced intraoperative blood loss, lower need for additional uterotonics, and favorable uterine tone assessments. These findings support the potential of carbetocin as a reliable and safe alternative to oxytocin in high-risk deliveries. However, due to methodological constraints including single-center design, lack of blinding, and absence of cost-effectiveness data, these results should be interpreted with caution. Further large-scale, multicentric studies are necessary to validate these findings and to assess their applicability across diverse patient populations and healthcare settings.

## References

[1] Maged AM, Hassan AM, Shehata NA (2016). Carbetocin versus oxytocin for prevention of postpartum hemorrhage after vaginal delivery in high risk women. J Matern Fetal Neonatal Med.

[2] Huang X, Xue W, Zhou J, Zhou C, Yang F (2022). Effect of carbetocin on postpartum hemorrhage after vaginal delivery: a meta-analysis. Comput Math Methods Med.

[3] Jin XH, Li D, Li X (2019). Carbetocin vs oxytocin for prevention of postpartum hemorrhage after vaginal delivery: a meta-analysis. Medicine (Baltimore).

[4] Gallos I, Williams H, Price M (2019). Uterotonic drugs to prevent postpartum haemorrhage: a network meta-analysis. Health Technol Assess.

[5] Bekkenes ME, Fagerland MW, Solberg OG, Aaberge L, Klingenberg O, Norseth J, Rosseland LA (2022). Exploring cardiac effects after oxytocin 2.5 IU or carbetocin 100 μg: a randomised controlled trial in women undergoing planned caesarean delivery. Eur J Anaesthesiol.

[6] Abdel Fatah MA, Salama KM, Edris YM, Metwally AM (2022). Carbetocin versus oxytocin in the prevention of postpartum hemorrhage following vaginal delivery in high risk patients. Benha Med J.

[7] van der Nelson H, O'Brien S, Burnard S (2021). Intramuscular oxytocin versus Syntometrine(®) versus carbetocin for prevention of primary postpartum haemorrhage after vaginal birth: a randomised double-blinded clinical trial of effectiveness, side effects and quality of life. BJOG.

[8] Theunissen FJ, Chinery L, Pujar YV (2018). Current research on carbetocin and implications for prevention of postpartum haemorrhage. Reprod Health.

[9] Dahlke JD, Mendez-Figueroa H, Maggio L, Hauspurg AK, Sperling JD, Chauhan SP, Rouse DJ (2015). Prevention and management of postpartum hemorrhage: a comparison of 4 national guidelines. Am J Obstet Gynecol.

[10] Escobar MF, Nassar AH, Theron G (2022). FIGO recommendations on the management of postpartum hemorrhage 2022. Int J Gynaecol Obstet.

[11] World Health Organization (2012). WHO Recommendations for the Prevention and Treatment of Postpartum Haemorrhage.

[12] Al Zubaidi S, Alhaidari T (2022). Heat stable carbetocin vs. oxytocin for the prevention of post-partum hemorrhage in emergency caesarean delivery: a randomized controlled trial. J Perinat Med.

[13] Hunter DJ, Schulz P, Wassenaar W (1992). Effect of carbetocin, a long-acting oxytocin analog on the postpartum uterus. Clin Pharmacol Ther.

